# Suppression of autophagy by chloroquine sensitizes 5-fluorouracil-mediated cell death in gallbladder carcinoma cells

**DOI:** 10.1186/2045-3701-4-10

**Published:** 2014-03-03

**Authors:** Xiao Liang, JiaCheng Tang, YueLong Liang, RenAn Jin, XiuJun Cai

**Affiliations:** 1 Key Lab of Surgery of Zhejiang province, Sir Run Run Shaw Hospital, Zhejiang University, Hangzhou 310016, People’s Republic of China

## Abstract

Autophagy1 is a complex of adaptive cellular response that enhances cancer cell survival in the face of cellular stresses such as chemothery. Here we show that in human gallbladder carcinoma (GBC) cells lines, SGC-996 and GBC-SD, autophagy is induced by the DNA damaging agent 5-fluorouracil (5-FU). While in combination with the pre-treatment of chloroquine (CQ), a inhibitor of autophagy, the inhibition of 5-FU to the proliferation and viability of GBC cells was potentiated. Furthermore, 5-FU treatment resulted in a general increase of the apoptotic rate and G0/G1 arrest of GBC cells, and the effect was potentiated by CQ pre-treatment. Since *5*-*FU induced autophagy in GBC cells*, and CQ inhibited autophagy, our findings suggest a possible mechanism that CQ inhibited 5-FU-induced autophagy, which modified the cytotoxicity of 5-FU. The combination therapy of CQ and 5-FU should be considered as an effective strategy for the treatment of gallbladder carcinoma.

## Introduction

To improve cancer cure rates, understanding of the mechanisms of the anticancer agents, as well as the mechanisms of acquisition of chemoresistance by cancer cells, is essential. Primary gallbladder carcinoma is one of the most common malignancies of the digestive tract in china and has been increasing incidence worldwide [1]. There is no specific symptom for such patients. In the majority of cases, the diagnosis of this carcinoma is usually made postoperatively on tumors at an advanced stage, resulting in a 5-year survival rate of <10% and almost half of patients already have metastatic disease at the time of surgery [2,3]. So far as we know, there are no adjuvant chemotherapeutic combinations widely accepted for the primary gallbladder carcinoma due to their toxicity, drug resistance and limited efficacy. One approach to overcome this major problem may be the discovery of new therapeutic applications for already existing drugs, which is termed ‘repurposing’ [4].

CQ, a widely used antimalaria drug, has been used for six decades as its effectiveness, low price, low toxicity to humans and well understood pharmacological properties [5,6]. CQ is also a choice for treatment of diverse diseases such as rheumatoid arthritis, lupus erythematosus and amoebic hepatitis [7,8]. More recently, importance has been attached to the ability of CQ to block autophagy by inhibiting lysosomal proteases and autophagosome-lysosomal fusion events [9]. Since autophagy is thought to act as a cell-survival pathway in cancer [10,11], CQ has been studied as a potential agent in cancer therapy. It’s notably that combing CQ with the DNA alkylating agent cyclophosphamide significantly increased the rate of tumor regression and delayed tumor recurrence [12]. Up to now, CQ and its derivatives are the only inhibitors of autophagy available for clinical treatment of patients. There are more than twenty clinical trials listed on the clinicaltrials.gov website [13] using CQ or its derivatives to test if inhibition of autophagy in a clinical setting can increase the effectiveness of cancer therapies [14].

Autophagy is a highly conserved survival response to growth limiting conditions, such as nutrient depletion, hypoxia and the presence of cytotoxic drugs [15,16]. It is genetically regulated by a family of autophagy-related (*ATG*) genes and can be detected by molecularly antibody-based detection of gene, microtubule-associated protein 1 light chain 3 (*LC3*). LC3 is constitutively expressed at low levels in most cells, and conjugated with phosphatidylethanolamine targets the autophagosomal membrane. The conjugated form of LC3 is called LC3-II and regarded as specific marker of autophagy [17]. Meanwhile, recent studies indicate the p62 protein function as an adaptor molecule involved in activating autophagy that interacts with polyubiquitinated protein aggregates and targets them to autophagosomes [18].

In the present study, we aimed to investigate the effects of the combination of chemotherapy with CQ on two kinds of gallbladder-carcinoma-derived cells, namely SGC-996 and GBC-SD. 5-FU is one of the major antitumor agents widely used against cancer for about 40 years. It exerts its anticancer effects through the inhibition of thymidylate synthase and the incorporation of its active metabolites, into RNA and DNA so as to influence the uracil metabolism *and has been used in Phase II trial of combination chemotherapy for advanced cancers of the gallbladder*[19,20]. Our research reveals the chemosensitizer of CQ on 5-FU may be partly dependent on its ability to inhibit autophagy. Moreover, 5-FU-induced apoptosis was enhanced after the inhibition of autophagy, suggesting a novel and promising strategy to increase the clinical efficacy of 5-FU for the treatment of gallbladder carcinoma.

## Materials and methods

### Reagents and antibodies

5-FU, CQ and bovine serum albumin (BSA) were purchased from Sigma-Aldrich. RPMI-1640, DMEM medium and fetal bovine serum (FBS) were from Gibco. Primary antibodies against LC3, GAPDH were from Cell Signaling Technology, Inc. Primary antibodies against P62, Atg5, Atg7 were from Epitomics, Inc. The GFP-LC3 plasmid was a gift from Dr. Hong-Chuan Jin’s lab at Zhejiang University, China.

### Cell cultures and transfection

Human gallbladder carcinoma cell line GBC-SD was bought from cell bank (Chinese Academy of Sciences). Each respectively, SGC-996 or GBC-SD cells was maintained in RPMI-1640 or DMEM supplemented with 10% (v/v) FBS and 1% (v/v) penicillin/streptomycin and incubated in a humidified 5% CO_2_ incubator at 37°C. The plasmids (GFP-LC3) or small interfering RNA (siRNA; Atg5 and Atg7) were transiently transfected into cells with Lipofectamine 2000 transfection or RNAi MAX reagent according to the manufacturer’s instructions (Invitrogen). After 24 hours, the cells were treated with 5-FU or CQ and subjected to fluorescent analysis or Western blotting assay. The SGC-996 cell line was provided by Dr. Ying-Bin Liu’s lab at Xin Hua Hospital Affiliated to Shanghai Jiao Tong University School of Medicine, China.

### FU and CQ treatment

Two human GBC cells were seeded and grown until they reached about 40-50% subconfluence. And then the cells were pre-treated with CQ for 12 hours, after washing with PBS the cells were treated with or without 5-FU for 48 h. The treatment was washed and replaced with regular media. Since 100 μM CQ mostly induced the formation of Acidic vesicular organelles (AVOs) while did minimal inhibition on GBC cells in 12 hours, in the subsequent experiments, the dose of CQ was set at 100 μM, followed by washing with PBS and then treated with 5-FU (5 μM) for another 24-48 h.

### Cytotoxicity assay

The cytotoxicity of chemicals against SGC-996 and GBC-SD cells was determined by CCK-8 assay. Cells were seeded into 96-well plates and treated with chemicals with different concentrations. After 24 h or 48 h incubation, 20 μl CCK-8 was added into each well for 4 h incubation. The absorbance was then measured using a model ELX800 Micro Plate Reader (Bio-Tek Instruments, Inc.) at 450 nm.

### Detection of acidic vesicular organelles (AVOs)

Cells undergoing autophagy generally develop double-membraned, acidic vesicular organelles (predominantly of autophagosomes and autolysosomes), which can be detected by specific dyes. Acridine orange (AO) is a fluorescent *emit green light when it bounds to DNA*, while it accumulates in acidic spaces and fluoresce bright red. *It selectively recognize autophagosomes and autolysosomes*, and the intensity of the red fluorescence is proportional to the degree of acidity, also represents AVOs formation [21]. SGC-996 and GBC-SD cells were prepared and treated as described, and the cells were resuspended in PBS (−) and stained with AO (5 μg/ml) for 15 min at room temperature. The cells were examined under a fluorescence microscope at 40 × objective lens magnification.

### Cell mortality analysis

1 × 10^5^ cells were prepared and treated as described, collected by trpsinization, centrifuged, resuspended in 500 μl PBS and stained with 0.5% trypan blue. The unstained cells were quantified using a counting chamber.

### Apoptosis detection

1 × 10^5^ cells were prepared and treated as described, collected by trpsinization, centrifuged, washed twice with 3 ml PBS, resuspended in 500 μl PBS and stained with 1% Annexin V-FITC/PI, analyzed by FACS caliber (Becton Dickinson).

### Cell cycle analysis

1 × 10^5^ cells were prepared and treated as described. After serum starved starvation and treatment, cells were harvested, washed once with 3 ml PBS, centrifuged, resuspended in 1 ml PBS and fixed with 80% methanol to obtain a final concentration of 70%-75%. The fixed cells were stored in a −20°C at least for 12 h. Before analysis, cells were washed once with 3 ml PBS, resuspended in 250 μl PBS containing 1% RNase and 1% propidium iodide. After incubation in dark for 30 minutes, treated cells were analyzed by FACS caliber and the obtained results were analyzed by the Cell Quest software.

### Colony forming assay

SGC-996 cells, suspended in fresh culture medium, were plated 500 cells/well onto 35 mm Dish. The viability cells were allowed to attach in 24 hours and treated with CQ at 100 μM for 12 hours, washed with PBS, and/or treated by 5-FU at 5 μM for 48 hours. Then, cells were washed with PBS (−), and fed with fresh culture medium, without CQ and/or 5-FU, and allowed to grow for 14 days in normal culture conditions (37°C, 5% CO_2_). To visualize colonies contained 50 or more cells during the 14 days of culture, media was removed, cells were fixed in 3.7% paraformaldehyde for 15 min and stained with crystal violet and the colonies were counted under light microscope. For each experimental condition, colonies were presented as the mean number ± SD from at least 3 independent experiments were counted.

### Protein isolation and western blots analysis

After treatment, cells were washed with PBS and lysed with RIPA buffer with protease inhibitors. Protein was quantitated using BCA protein assay (Pierce). 10–30 mg of total protein were resolved by SDS-polyacrylamide gel electrophoresis, transferred to a PVDF membrane (Millipore) and then detected by the proper primary and secondary antibodies before visualization with a chemiluminescence kit (Pierce). The visualization was done with Image Quant LAS-4000 (Fujifilm, Tokyo, Japan).

### Fluorescence microscopy

Cells were transfected with GFP-LC3 plasmids, followed by treatment as described. The cells were then rapidly washed with PBS and fixed at room temperature for 15 minutes with 3.7% paraformaldehyde. After being washed with PBS twice, cell nuclei were stained by DAPI. Samples were observed under a fluorescence microscope.

### Transmission electron microscopy

Treated cells were washed and fixed for 30 min in 2.5% glutaraldehyde. The sample were post-fixed in 1.5% osmium terroxide, dehydrated in ascending grades of ethanol solutions (50%, 70%, 90%, 100%) and acetone, prior to embedding in araldite resin. Thin sections were prepared on an ultramicrotome and stained with uranyl acetate and wolfberry lead acid. All sections were examined and photographed with a Philips TECNAI 10 electron microscope at 80 kV.

### Statistical analysis

Unless otherwise stated, data was expressed as the mean ± SD and analyzed by Student’s t test, differences were considered significant when the *P* value was less than 0.05.

## Results

### Effect of 5-FU and CQ on the proliferative activity of GBC cells

The CCK-8 assay revealed CQ show a weak cytotoxic effect *at the dose of 100 μM* for 12 hours *while the cytotoxicity was significantly increased by 24 h*-*treatment of the same concentration* (Additional file 1: Figure S1A). On the other hand, 100 μM CQ mostly induced the formation of AVOs equal to the dose of 200 μM (Additional file 1: Figure S1B), with minimal inhibition on GBC cells at the same time. According to above results, the concentration of 100 μM of CQ in 12 h-treatment which show slight inhibition on GBC cells were selected for the further experiments.

### CQ blocked autophagy induced by 5-FU in GBC cells

**Figure 1:**
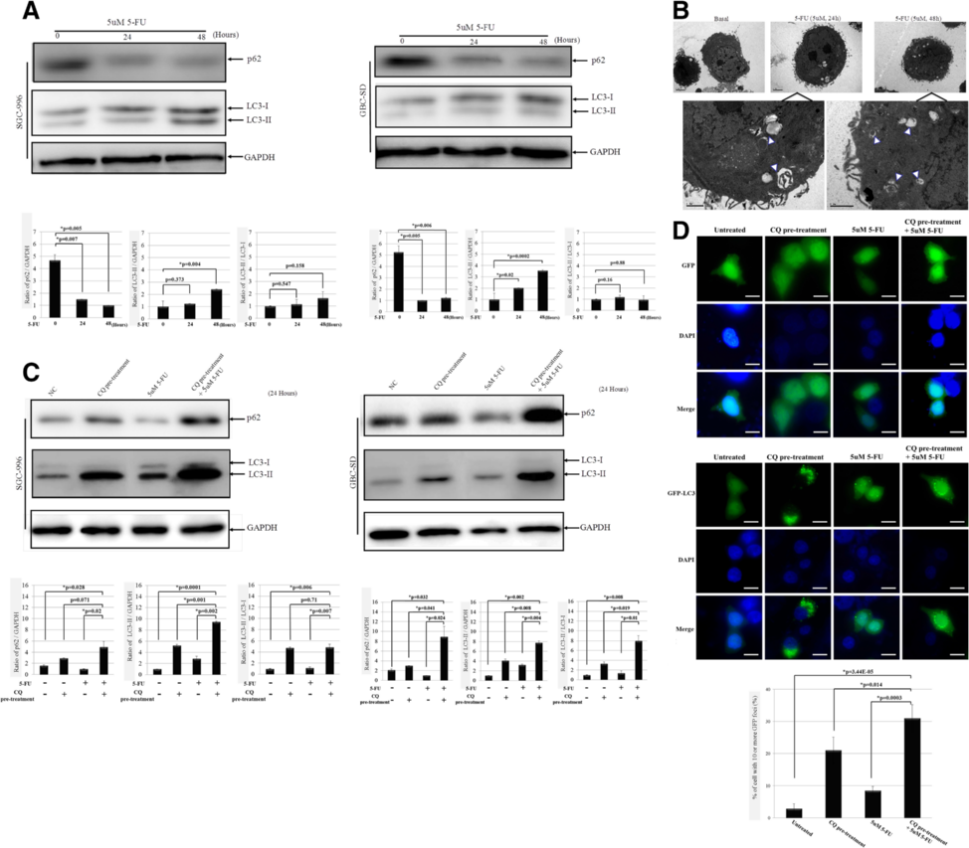
**CQ increased the expression of LC**-**3 and p62 in GBC cells after treatment with 5**-**FU. (A)** Time course detection of LC-II and p62 following treatment of GBC cells with 5-FU for 24 hours or 48 h by western blot. The lower panels represent the densitometric values obtained from the bands of the western blot (*, p < 0.05 vs. control, n = 3). **(B)** Representative electron microscopy images of control SGC-996 cells (upper left), 5-FU-treated (5 μM) for 24 hours (upper middle) or 48 hours (upper left). Obvious autophagic vacuoles were highlighted by white arrows in higher magnification (lower), with typical double-layer membrane containing organelle remnants present in 5-FU treated cells rather than untreated cells. **(C)** Western blot analysis of LC-II and p62 from lysates of GBC cells treated with 5-FU alone for 48 h or after 12 hours pre-treatment with CQ. GAPDH was used as a loading control and the expressions of autophagy-related proteins (LC3-II, p62) were quantified (*,p < 0.05 vs. control, n = 3). **(D)** SGC-996 cells were transfected with vectors expressing either GFP or GFP-LC3, followed by pre-treatment of 100 μM CQ and/or 5 μM 5-FU as described. The GFP or GFP-LC3 staining patterns were analyzed by fluorescence microscopy. The GFP control cells display diffuse GFP distributed throughout the cytoplasm, coincident with GFP-LC3 patterns of SGC-996 vehicle control cells (lower left panels). Both 5-FU and CQ (lower middle panels) induced punctuate patterns in GFP-LC3 patterns, while the latter was more bright and clear. 5-FU combined with CQ (lower right panels) showed a similar diffuse GFP-LC3 but notably bright punctuate patterns. Images are representative of at least three independent experiments and quantification of green dots is shown in graph as mean ± SD (*,p < 0.05, n = 5, Scale bar = 10 μm).

*In order to investigate the effect of 5*-*FU on autophagy* as well as the inhibitory effect of CQ, the expression of LC3-II and p62 in GBC cells was investigated by Western blot (Additional file 2: Figure S2). Since earlier reports have demonstrated that the antitumor effects of 5-FU depend on exposure duration rather than plasma concentration levels [22], the time course following treatment of GBC cells with 5-FU alone was *conducted*. The results revealed a time-dependent *changes of the autophagic markers*, *including* accumulation of LC3-II and *degradation of p62* (Figure 1A). *More importantly*, CQ pre-treatment *markedly increased both LC3*-*II and p62 protein levels*, *indicating the enhanced autophagic flux induced by 5*-*FU in GBC cells* (Figure 1C). Consistently, the ultrastructural features of SGC-996 cells, following 24-h or 48-h treatment with 5-FU (5 μM), revealed *morphological changes including obvious autophagic vacuoles* in the cytoplasm compared with cells in basal state (Figure 1B).Moreover, green fluorescence showed mostly a uniform distribution in untreated GFP-LC3 expressing SGC-996 cells. Coincidentally, a few green dots were observed under 5-FU treatment conditions and punctuate patterns of GFP-LC3 representing autophagic vacuoles were formed in the cytoplasm after treatment of 5-FU combined with CQ (Figure 1D). These results showed that 5-FU induced the autophagy activation and autophagy process occurred within several hours after treatment with drug.

### CQ potentiated the suppression of the growth in GBC cells induced by 5-FU

**Figure 2:**
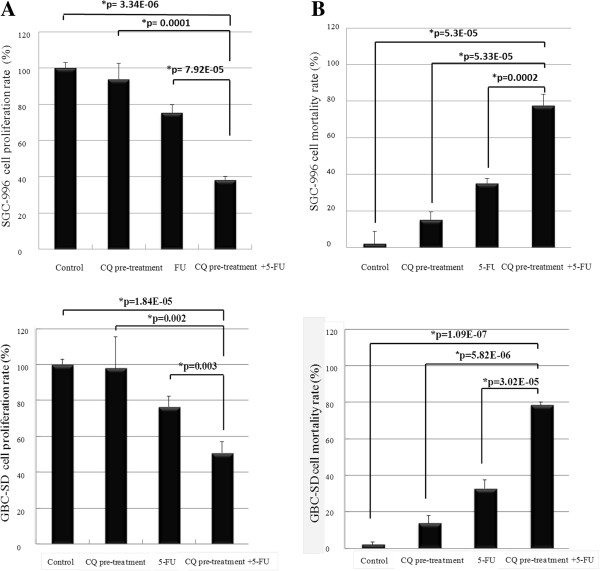
**Effect of CQ in 5**-**FU**-**induced GBC cell proliferation and mortality inhibition.** The effect of CQ-pretreatment on the inhibitory effect of 5-FU on the proliferative activity and cell mortality rate of SGC-996 cells and GBC-SD cells was investigated by CCK-8 assay and trypan blue exclusion staining. The y-axis represents the proliferation/mortality rate, calculated as the ratio to normal control (untreated cells). Values were given as mean ± SD. **(A)** Pre-treatment of cells with CQ at 100 μM for 12 hours prior to exposure to 5-FU at 5 μM for 48 hours, resulted in significantly potentiation of the inhibitory effect (25% vs. 62% inhibition (SGC-996 cells) and 24% vs. 50% inhibition (GBC-SD cells) for 5-FU alone and CQ + 5-FU, respectively) (*,p < 0.05 vs. control, n = 5). **(B)** When cells were treated with CQ at 100 μM for 12 hours prior to exposure to 5-FU at 5 μM for 48 hours, the mortality rate reduction was notably potentiated (60% vs. 28% live cells (SGC-996 cells) and 67% vs. 21% live cells (GBC-SD cells) for 5-FU alone and CQ + 5-FU , respectively) (*,p < 0.05 vs. control, n = 5).

Our studies demonstrated that 5-FU inhibited the proliferation of GBC cells in time- and dose-dependent maner. Meanwhile, a single dose of 5-FU at 5 μM was required to reduce around 30% proliferative rate in GBC cells according our experiments and below the maximum concentration to cause the myelotoxicity. After a pre-treatment of 100 μM CQ for 12 hours, which had nearly no inhibitory effect on GBC cells, notably potentiated over 50% suppress proliferation effect of 5 μM 5-FU treatment for 48 hours (Figure 2A). Similar to the results of cell mortality analysis, the growth of GBC cells were significantly decreased by combination treatment of CQ and 5-FU, in comparison with the 5-FU or CQ alone (Figure 2B).

### CQ enhanced the cytotoxicity of 5-FU through inhibiting autophagy

Since autophagy is a mechanism to promote or delay cell death [23], we assessed whether inhibition of autophagy contributed to the enhanced cytotoxicity of 5-FU when combined with CQ. Moreover, we also found 3-MA (a well-know autophagy inhibitor) potentiated the suppression of the growth in GBC cells induced by 5-FU (data not show). It’s supposed the resistance of GBC cells to 5-FU may be overcome with autophagy inhibitor.

**Figure 3:**
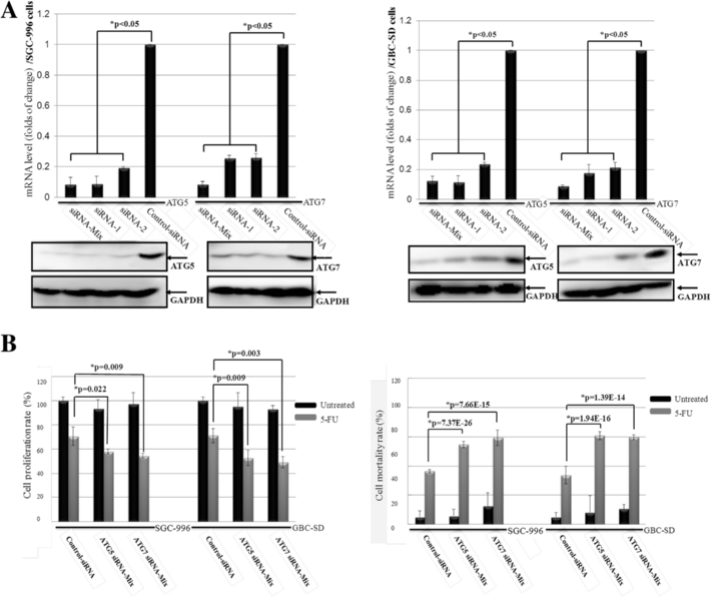
**CQ enhanced the cytotoxicity of 5**-**FU through inhibiting autophagy. (A)** The contribution of autophagy to SGC-996 and GBC-SD cells after treatment with 5-FU was examined by ATG5 and ATG7 with siRNA. The mRNA expression of each gene were significantly reduced by siRNA treatment (upper), in parallel with the protein levels after the transfection (lower). GAPDH was used as a loading control. **(B)** GBC cells transiently transfected with negative control siRNA, Atg-5 or Atg-7 siRNA were treated with 5 μM 5-FU for 48 hours. Cell proliferation and mortality rates were measured by CCk-8 assay and the trypan blue exclusion staining (*,p < 0.05 vs. control, n = 3).

Two key regulators of autophagy, ATG5 and ATG7 with short interfering RNA (siRNA) were designed to examine the contribution of autophagy to survival and recovery of GBC cells after the treatment of 5-FU. The levels of knockdown achieved for each gene mRNA and protein expression, were mostly great than 80% at 72 hours (Figure 3A). 24 hours after addition of siRNA, cells were treated with 5 μM 5-FU for 48 hours. The adherent cells were collected, stained with trypan blue and counted (Figure 3B). These cells counts indicated that knockdown of *ATG5* or *ATG7* reduced the proliferation and mortality at 48 h post treatment with 5-FU at concentration of 5 μM. Taken together, these data suggest that as the specific inhibitor, CQ enchanced the cytotoxicity of 5-FU by inhibiting autophagy.

### CQ increased apoptosis and potentiated the G0/G1 arrest of GBC cells induced by 5-FU

**Figure 4:**
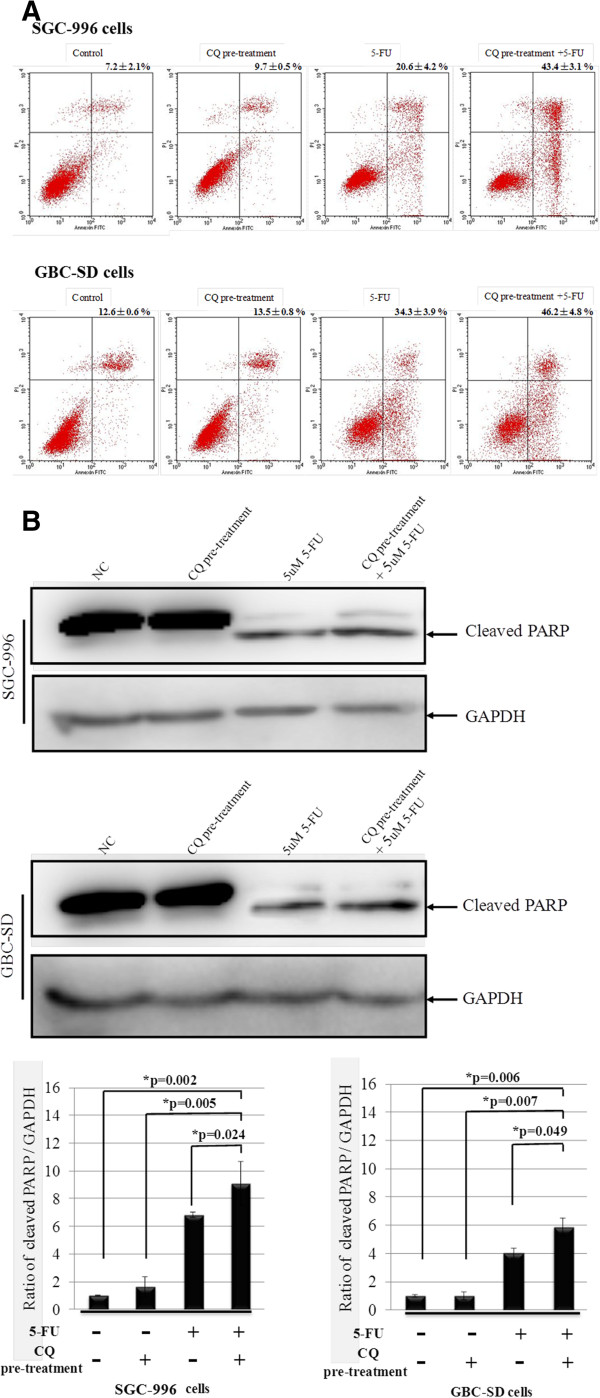
**CQ potentiates apoptosis of SGC cells induced by 5**-**FU. (A)** The population of annexin V^+^ apoptotic cells were evaluated by FCM using annexin V-FITC/PI staining in SGC-996 cells and GBC-SD cells after CQ-pretreatment at 100 μM for 12 hours, followed by 5-FU at 5 μM for 48 hours. The percentage of annexin V^+^ apoptotic cells increased by pretreatment with CQ followed by 5-FU. Values were given as mean ± SD. **(B)** GBC cells were treated with CQ at 100 μM for 12 hours, washed with PBS, and/or treated by 5-FU at 5 μM for 48 hours respectively. GAPDH was used as an internal control. Cleavage of PARP induced by 5-FU and CQ in GBC cells was examined with Western Blotting analysis. The values of the band intensity below the figure represent the densitometric estimation of each band normalized by GAPDH. Experiments were repeated three times with reproducible results.

In clarify whether the inhibitory effect of 5-FU combined with CQ on GBC cells was due to apoptosis and/or cell growth arrest, flow cytometry and colony formation assay were used. CQ pre-treatment resulted increasing of the percentage of apoptotic cells followed by 5-FU treatment (34.3% compared to 46.2% for treated with 5-FU alone and CQ + 5-FU in GBC-SD cells, 23.4% compared to 48.6% for 5-FU alone and CQ + 5-FU in SGC-996 cells, respectively) (Figure 4A). *Consistently*, *the level of cleaved product of caspases substract Poly ADP*-*ribose Polyermerase* (*PARP*) *was correlated with the activation of caspases* (Figure 4B).

**Figure 5:**
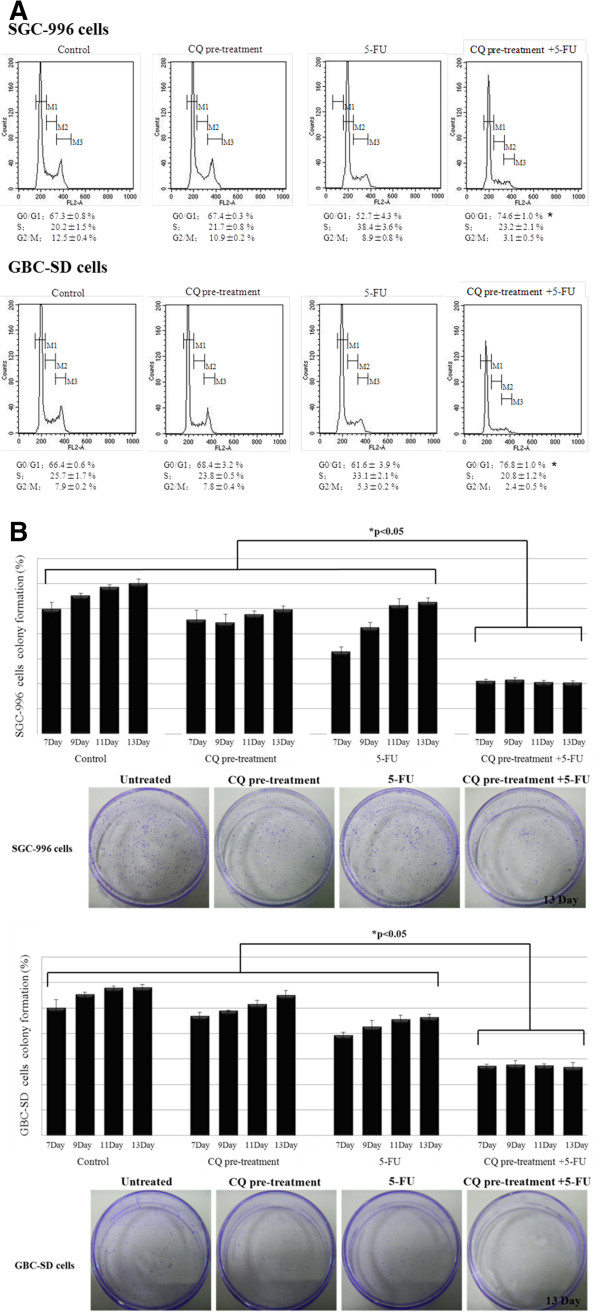
**CQ inhibited the colony formation of GBC cells combined with 5**-**FU. (A)** The analysis of changes in cell cycle was quantified by flow-cytometry after PI staining in SGC-996 cells and GBC-SD cells treated without or with 5-FU for 48 h after 12 hours pre-treatment without or with CQ. Values were given as mean ± SD. Treatment of GBC cells with 5-FU alone resulted in increased intra-S arrest, but the G1 arrest was potentiated by CQ-pretreatment. Meanwhile, G2/M progression of GBC cells was blocked by the treatment with 5-FU alone, and it was potentiated by the 12 hours pretreatment of CQ. **(B)** The colony formation assay was used to assess recovery GBC cells treated without or with 5-FU for 48 h after 12-h pretreatment without or with CQ (100 μM). Values were given as mean ± SD. Single treatment of 5-FU or CQ alone resulted in a delay and slight inhibition of the colony-forming ability, at day 13 of culture most cells were recovery and had formed colonies. However, pre-treatment of cells with CQ prior to exposure to 5-FU resulted in enhanced inhibitory effect on the colony forming ability, which was recovery lower than 50% of control untreated cells at day 13 (*, p < 0.05 vs. control, n = 3).

Moreover, pre-treatment with CQ resulted in increment of *the percentage of GBC cells at the G0*/*G1 phase*, compared with the cells treated with 5-FU alone (52.7% compared to 74.6% for 5-FU alone and CQ + 5-FU in SGC-996 cells, 61.6% compared to 76.8% for treated with 5-FU alone and CQ + 5-FU in GBC-SD cells, respectively) (Figure 5A). The viability of the GBC cells after treatment with 5-FU and/or CQ was assessed by the colony formation assay. Cell were pre-treated with or without CQ for 12 hours followed by 5-FU treatment for 48 hours, and then fed with fresh complete culture medium for 2 weeks. Single treatment of 5-FU or CQ caused a delay and slight inhibition of the colony formation, whereas pre-treatment of cells with CQ at 100 μM for 12 hours prior to 5-FU significantly reduced colony formation (Figure 5B).

## Discussion

To our best knowledge, it is the first report to show the potential applicability of CQ to improve the cytotoxicity of 5-FU in SGC-996 and GBC-SD cells. The aim of the research is to investigate the effect of 5-FU on human gallbladder carcinoma cells by CQ, the well-known lysosomotropic agent and the inhibitor of autophagy.

Since previous studies have demonstrated that CQ does cytotoxic effects to certain cancer cell [24,25], we determined the dose of CQ to mostly inhibit the autophagy without a direct cytotoxic effect on GBC cells. Previous studies have indicated that the biological effect of CQ is concentration-dependent. When the concentration increasing, CQ inhibits cell growth and induces vacuolation with acidic compartments. At higher concentrations, or over longer periods, CQ directly induces apoptosis and necrosis. In this study, CQ showed a weak cytotoxic effect at the dose of 100 μM for 12 hours, the proliferation rate in such condition is about 95% compared to the normal control. Therefore, the dose we used for this research did not have a direct cytotoxic effect on GBC cells.

*Among the chemotherapeutic agents used against cancer*, *5*-*FU remains the popular one. The molecular mechanisms of 5*-*Fu*-*induced autophagy activation are complicated. In colon cancer cell*, *autophagy takes part in the response to 5*-*FU through the regulation of Bcl*-*xL protein*[26], *it appears to be a link between autophagy and the apoptosis pathways. On the other hand*, *p53*-*AMPK*-*mTOR may participate in 5*-*FU*-*induced autophagy response as well*[27]. Here we showed that combinational treatment of CQ and 5-FU had better efficacy in killing GBC cells. Differing from other inhibitors of autophagy, CQ inhibit autophagy at the time of autophagosomes have already been formed, we observed CQ accumulated AVOs in a concentration-dependent maner. Besides, the expression of LC3-II is time- and dose-dependent as well, which was in parallel with the results of AVOs, indicating CQ blocked the degradation of autophagic vesicles and therefore the completion of autophagy. The treatment of GBC cells with combination of CQ and 5-FU resulted in potentiation of the inhibitory effect on the proliferation, viability and increasing rate of apoptotic cells as well. The colony formation assay was conducted to assess the morphologically distinction between the cells treated with CQ and/or 5-FU, single treatment of 5-FU or CQ alone resulted in a delay and partially inhibition on colony-forming ability, suggest that autophagy is a mechanism necessary for cell survival under such conditions, and result GBC cells to a temporary quiescent state which probably dependent on the cell arrest to G0/G1 phase. While the combination of CQ pre-treatment and 5-FU significantly inhibited the colony-forming ability of GBC cells, and was not restore after 13 days in normal culture. Our results are consistent with other reports that autophagy inhibition by CQ or other autophagy inhibitor (e.g., ATG-5 siRNA and ATG-7 siRNA) induces cell death in cancer cell types [28-34].

Treatment of the GBC cells with 5-FU results the increase of LC3-II and decrease of p62 expression compared with the control untreated cells, which was time-dependent. While it’s convinced that autophagy can be inhibited by CQ, we hypothesized that GBC cells induced autophagy as the defense mechanism against 5-FU, and the inhibition of autophagy treated by CQ could be responsible for the potentiation of the cytotoxicity of 5-FU. The siRNAs specific to human Atg5 and Atg7 were used to block the autophagy at a proximal step as ATGs are essential to the formation of the Atg-Atg12 complex to activate autophagy. We examined the proliferation and mortality rates of the GBC cells treated with siRNA and/or 5-FU, the results of siRNA-mediated knockdown assays revealed a lack of the ability of autophagy can significantly enhance the efficacy of 5-FU on GBC cells and provided an opportunity for human gallbladder carcinoma.

*Recently*, *autophagy has been shown to play a role as self*-*defense mechanism in promoting tumor cell resistance to the chemotherapy. Howerver*, *the mechanism remains debated. In this study*, *we demonstrated that autophagy may contribute to chemoresistance in GBC cells*, *since pre*-*treatment of CQ increased the 5*-*FU*-*induced apoptosis and the G0*/*G1 arrest in vitro. The relationship between autophagy and apoptosis is quite complicated. In some case they had no connection*[35,36]*while some report demonstrated autophagy might promote or even restrain apoptosis*[37,38]. *At the molecular level*, *the interaction between them is manifested by numerous genes including Atg5*, *the Bcl*-*2 family*, *p53*, *ARF*, *DAPk*, *and E2F1*[39-42]. *The crosstalk between apoptosis and autophagy is a key factor in the outcome of cancer*[42]*while how autophagy helps tumor cells resist to apoptosis remains poorly defined. Similarly*, *we also observed inhibition of autophagy enchanced 5*-*FU*-*induced cell growth. Since pre*-*treatment with CQ resulted in increment of the percentage of GBC cells at the G0*/*G1 phase in our present study*, *it is possible that cell cycle influences autophagic degradation*, *and inhibition of autophagy may lead cells to be arrested to the G0*/*G1*-*phase. While the exact mechanism for inhibition of autophagy increase the cytotoxicity of 5*-*FU in GBC cells deserved to be verified*.

In summary, here we report, for the first time, that 5-FU induced cytotoxicity can be potentiated by CQ pre-treatment. Since we showed that blocking of autophagy by genetic (ATG5 siRNA and ATG7 siRNA) or pharmacological (CQ) means induced cell death in GBC cells grown with 5-FU, it’s possible that autophagy plays a protective role in proteasome inhibitor-induced cell death by elimination cytotoxic cellular component, it may be an resistant factor which diminishes therapeutic effect in both sensitivities and resistantance of gallbladder carcinoma. We therefore propose that blocking autophagy simultaneously can overcome resistance of GBC cells to 5-FU-induced cell death. Further study, for example, in pre-clinical trial using animal models of gallbladder carcinoma is required to test the efficacy and efficiency of CQ and 5-FU *in vivo*.

## Competing interest

The authors declare that they have no competing interests.

## Authors’ contribution

XL participated in the experiments and drafted the manuscript. JCT carried out the experiments and prepare the Results and the Discussion sections of the manuscript. YLL participated in the experiments. RAJ analysed the data. XJC designed the study and directed its implementation. All authors read and approved the final manuscript.

## Supplementary Material

Additional file 1: Figure S1 Effect of CQ and 5-FU on the proliferative and growth activity of GBC cells. (A) The cell proliferative and growth activity of CQ-treated SGC-996 cells and GBC-SD cells for 12 and 24 hours were assessed by the CCK-8 assay and the trypan blue exclusion staining. The y-axis represents the proliferation rate or viability, calculated as the ratio to normal control (untreated cells). CQ treatment at 100 μM for 24 hours resulted in significant inhibition of the proliferative activity of SGC-996 cells and GBC-SD cells *comparing to the 12 hours treatment*, but not at lower doses (*, *p* < *0.05*, *n* = *3*) (B) The cell proliferative and growth activity of 5-FU-treated SGC-996 cells and GBC-SD cells for 24 and 48 hours were assessed by the CCK-8 assay and the trypan blue exclusion staining. The y-axis represents the proliferation rate or viability, calculated as the ratio to normal control (untreated cells) (*, p < 0.05, n = 3). (C) CQ induced the formation of AVOs of GBC cells. The formation of AVOs (*proportional to the intensity of red stain*) was obtained with the fluorescence microscopic examination, confirming the autophagy induced by vary doses of CQ (0, 10, 50, 100, 200 μM). Click here for file

Additional file 2: Figure S2 Starvations treatment increased the expression of LC-3 in GBC cells. GBC cells were cultured in Earl’s balanced salt solution (EBSS; Gibco® 14155–063) for 3 hours to activate starvation-induced autophagy. Lysates were then prepared and analyzed by immunoblotting using antibodies against various proteins. GAPDH was used as a loading control and both LC3-II/LC3-I and LC-3II/GAPDH densitometric ratios were marked. (*,p < 0.05 vs. control, n = 3). Click here for file
