# Supplementary figures and images for: Suppression of autophagy by chloroquine sensitizes 5-fluorouracil-mediated cell death in gallbladder carcinoma cells
Source: Cell Biosci. 2014 Mar 3;4:10. doi: 10.1186/2045-3701-4-10 (PMC4015784; doi:10.1186/2045-3701-4-10)

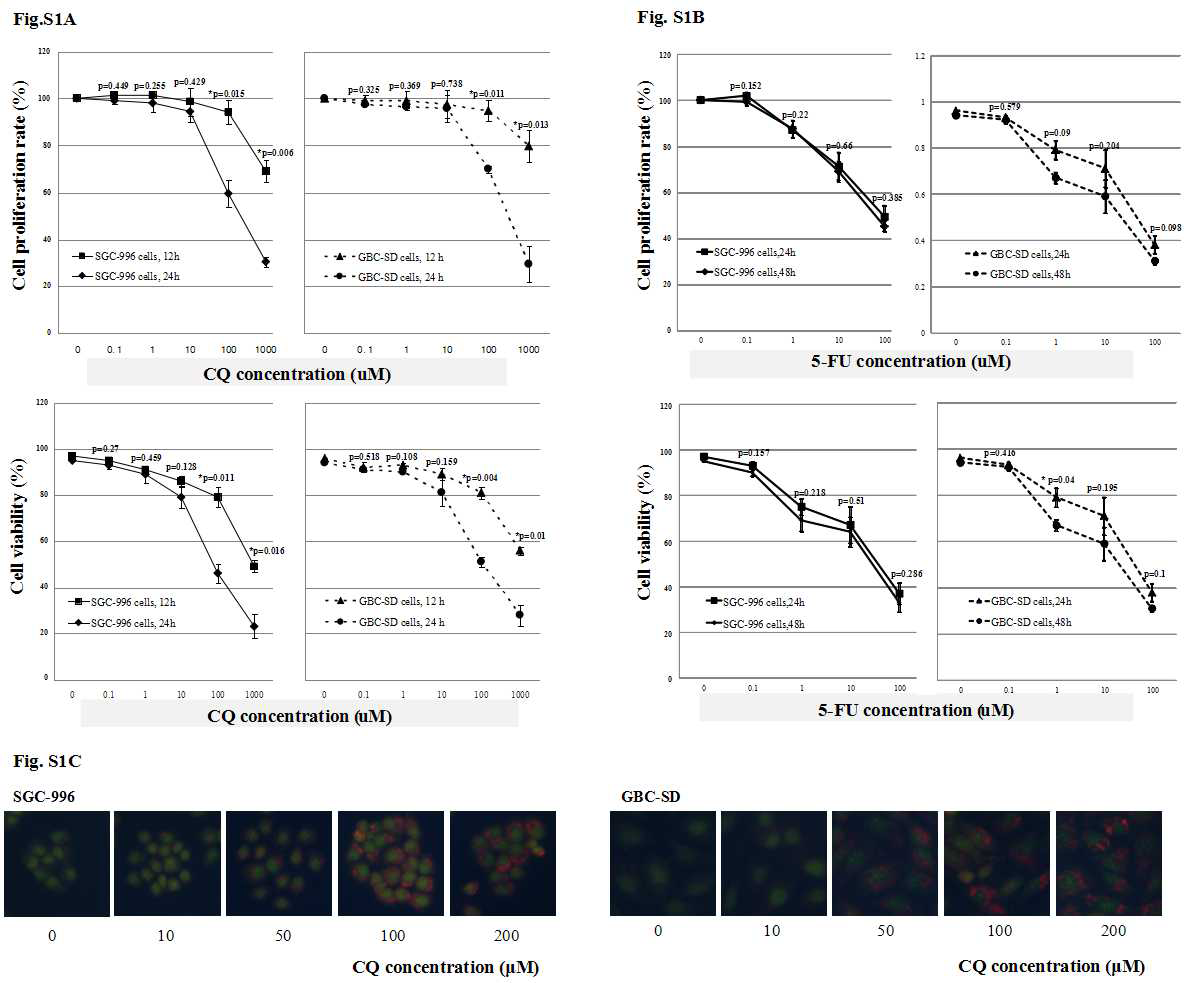

Supplement: Additional file 1: Figure S1 — Effect of CQ and 5-FU on the proliferative and growth activity of GBC cells. (A) The cell proliferative and growth activity of CQ-treated SGC-996 cells and GBC-SD cells for 12 and 24 hours were assessed by the CCK-8 assay and the trypan blue exclusion staining. The y-axis represents the proliferation rate or viability, calculated as the ratio to normal control (untreated cells). CQ treatment at 100 μM for 24 hours resulted in significant inhibition of the proliferative activity of SGC-996 cells and GBC-SD cells comparing to the 12 hours treatment, but not at lower doses (*, p < 0.05, n = 3) (B) The cell proliferative and growth activity of 5-FU-treated SGC-996 cells and GBC-SD cells for 24 and 48 hours were assessed by the CCK-8 assay and the trypan blue exclusion staining. The y-axis represents the proliferation rate or viability, calculated as the ratio to normal control (untreated cells) (*, p < 0.05, n = 3). (C) CQ induced the formation of AVOs of GBC cells. The formation of AVOs (proportional to the intensity of red stain) was obtained with the fluorescence microscopic examination, confirming the autophagy induced by vary doses of CQ (0, 10, 50, 100, 200 μM). [file 2045-3701-4-10-S1.jpeg]

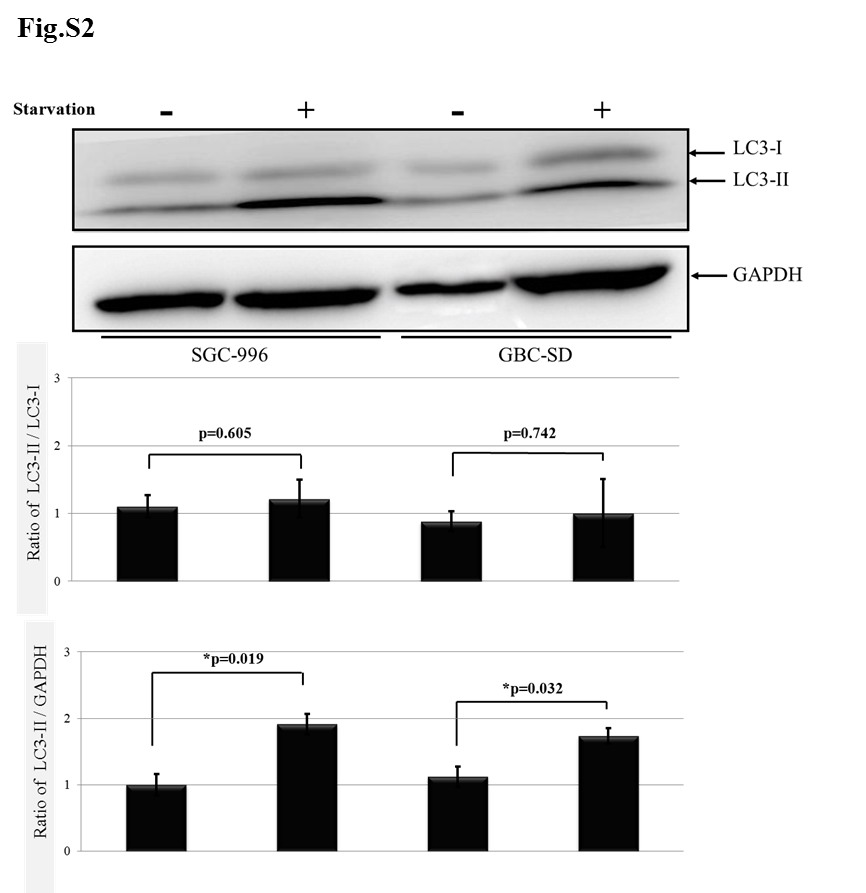

Supplement: Additional file 2: Figure S2 — Starvations treatment increased the expression of LC-3 in GBC cells. GBC cells were cultured in Earl’s balanced salt solution (EBSS; Gibco® 14155–063) for 3 hours to activate starvation-induced autophagy. Lysates were then prepared and analyzed by immunoblotting using antibodies against various proteins. GAPDH was used as a loading control and both LC3-II/LC3-I and LC-3II/GAPDH densitometric ratios were marked. (*,p < 0.05 vs. control, n = 3). [file 2045-3701-4-10-S2.jpeg]
